# Interest in Taking HIV Pre-exposure Prophylaxis Is Associated with Behavioral Risk Indicators and Self-Perceived HIV Risk Among Men Who Have Sex with Men Attending HIV Testing Venues in Sweden

**DOI:** 10.1007/s10508-020-01740-9

**Published:** 2020-06-03

**Authors:** Tobias Herder, Anette Agardh, Per Björkman, Fredrik Månsson

**Affiliations:** 1 Division of Social Medicine and Global Health, Department of Clinical Sciences, Malmö, Lund University, Jan Waldenströms gata 35, 214 28 Malmö, Sweden; 2 Clinical Infection Medicine, Department of Translational Medicine, Lund University, Malmö, Sweden

**Keywords:** Men who have sex with men (MSM), PrEP, HIV, HIV prevention, Sexual risk, Sexual orientation

## Abstract

This study explored factors associated with interest in taking PrEP among men who have sex with men (MSM) attending HIV testing venues in Sweden. Data from 658 HIV-negative respondents, surveyed by a questionnaire at six sites, were analyzed descriptively and by univariable and multivariable logistic regression. A total of 453 (68.8%) of the respondents expressed interest in taking PrEP. Reporting self-perceived risk of HIV acquisition as moderate or high, reporting ≥ 5 partners for condomless anal intercourse during the past year, and reporting hard drug use during the past year were independently associated with interest in taking PrEP. However, an aggregated variable of self-reported rectal gonorrhea, rectal chlamydia, or syphilis infection during the past year was not associated with interest in taking PrEP. Overall, Swedish MSM were well-informed regarding PrEP, and interest in taking PrEP was positively associated with sexual risk indicators.

## Introduction

In 2018, a total of 481 HIV infections were reported in Sweden (population 10.3 million, July 2019). While the majority of HIV infections were reported to have been contracted in another country, in 76 of the cases transmission had occurred in Sweden. Among the 76 cases, the most common route of transmission (51%) was sex between men. In all, 157 of the 481 new HIV cases were reported as transmission between men; a majority of them, 116, were reported to have contracted HIV abroad (Folkhälsomyndigheten, 2019).

Pre-exposure prophylaxis (PrEP) for HIV is effective in preventing HIV acquisition among men who have sex with men (MSM), both when used continuously and on-demand (Fonner et al., 2016; Grant et al., 2010; McCormack et al., 2016; Molina et al., 2015, 2017). In Sweden, the combination of the two antiretroviral drugs Tenofovir and Emtricitabine was registered for daily PrEP by the Swedish Dental and Pharmaceutical Benefits Agency in October 2016. The following year, Swedish recommendations concerning PrEP were published by the reference group for antiviral therapy (RAV). The recommendations list the following factors as indicators of increased risk of HIV infection and PrEP provision: MSM with two or more episodes of rectal gonorrhea and/or chlamydia, and/or lymphogranuloma venereum infection in the previous 2-year period and/or syphilis infection in the previous 5-year period. In addition, the recommendations note that information about drug use in conjunction with sex, selling sexual services, or trips to countries with high HIV incidence where unprotected sex is expected are factors that might indicate increased risk of HIV infection among MSM (RAV, 2017). However, the implementation of PrEP provision has been slow and uneven throughout the country, partly due to the regional autonomy of health care provision in Sweden. As stated in the recommendation from RAV, an expected 500 MSM in Sweden would be eligible for PrEP based on clinical indications (RAV, 2017), but the interest in the target population has been considerably higher. PrEP is currently prescribed by physicians at designated clinics, which has resulted in delays in treatment initiation among PrEP candidates. In October 2019, a waiting time of up to a year in Stockholm was reported, and although 435 MSM had started PrEP, an additional 600 MSM who had declared interest in starting PrEP had not yet received prescriptions (Johansson, 2019). In early 2020, media also reported that three MSM had seroconverted to HIV while waiting to for PrEP prescriptions in Stockholm (Thorén, 2020). Although there have been no major campaigns in Sweden promoting PrEP, the interest has been larger than expected.

Previous studies from various settings have found that awareness, interest, and willingness to take PrEP generally is high among MSM (Aghaizu et al., 2013; Goedel, Halkitis, Greene, & Duncan, 2016; Golub, Gamarel, Rendina, Surace, & Lelutiu-Weinberger, 2013; Hoagland et al., 2017; Iniesta et al., 2018; Kahle, Sullivan, & Stephenson, 2018; Werner et al., 2018) and that many MSM assess their need for PrEP appropriately (Bull et al., 2018; White et al., 2019). The identified studies were conducted either outside of Europe or among MSM in larger European cities, i.e., London, Berlin, and Madrid with higher HIV incidence and prevalence among MSM than in Sweden.

A handful of previous studies and reports have explored the prevention needs and preferences among Swedish MSM. Access to community-based testing, condom provision and MSM-specific clinics have been reported as preferred strategies (Folkhälsomyndigheten, 2015). In relation to PrEP we also need to consider the proportion of MSM who are not condom users and who have specific preventive needs (Berg, Tikkanen, & Ross, 2011) and that self-perceived risk affects HIV testing frequency (Persson et al., 2016a; Strömdahl, Liljeros, Thorson, Persson, & Forsberg, 2017) thereby also contact with clinics that prescribe PrEP. To our knowledge, no previous study has explored interest in taking PrEP among MSM in Sweden. Thus, the current study could contribute knowledge for the planning of expansion of PrEP provision in Sweden and in other similar settings.

The objective of this study was to explore if self-reported indicators for sexual risk behavior, self-perceived risk of HIV acquisition, PrEP knowledge, and self-reported history of sexually transmitted infections (STI) were associated with interest in taking PrEP for HIV prevention among HIV-negative MSM attending HIV testing venues in the three largest cities of Sweden.

## Method

### Participants

The current study analyzed data from a cross-sectional site-based survey of MSM attending HIV testing venues. The original study has ethical approval from the Regional Ethical Review Board in Lund, Sweden, and was commissioned to Lund University by the Public Health Agency of Sweden in 2018. Data were collected at three STI clinics within the public health care system and three community-based rapid HIV testing venues in the three most populous cities in Sweden (Stockholm, Gothenburg, and Malmö) between August and November 2018. At the time of the start of this study, PrEP was only prescribed for MSM in Gothenburg. During the data collection period, PrEP became available for MSM also in Malmö and Stockholm.

To reach the study population MSM, all men visiting the selected venues for HIV and/or STI testing were invited to answer a questionnaire, which consisted of a 33-item questionnaire covering sociodemographic background, interest and experiences of using PrEP, experiences and frequencies of HIV and STI testing, and sexual practices. Whereas all study venues target MSM, some of these also provide testing services for other groups (Table 1). When men visiting the venues registered for a scheduled appointment or for drop-in testing, they were given oral and written information about the study and a consent letter together with a questionnaire in either Swedish or English. At the three health care clinics, paper questionnaires were used while electronic questionnaires on tablets were used at the community-based venues. Men who chose to participate in the study had the opportunity to answer the survey while waiting for their appointment in the designated waiting area, and when needed, they were given the opportunity to finalize their questionnaire after their appointment. All the submitted questionnaires were collected at the study sites. The survey was anonymous and no patient information from the clinics was collected or linked to survey data.

**Table 1:** Participating HIV and STI testing venues and clinics where MSM were surveyed

Venue	City	Type (organization)	Questionnaire	Target group(s)	Data collection period
Venhälsan, Södersjukhuset	Stockholm	Public HIV/STI clinic (Region Stockholm)	Paper	MSM	Oct 8–Nov 30, 2018
Testpoint Stockholm	Stockholm	Community-based HIV testing venue (RFSL Stockholm)	Electronic	MSM and Trans persons	Sept 17–Nov 30, 2018
Könsmottagningen, Sahlgreska sjukhuset	Gothenburg	Public HIV/STI clinic (Västra götalandsregionen)	Paper	All men and women, with integrated MSM-clinic	Aug 27–Nov 30, 2018
Checkpoint Göteborg	Gothenburg	Community-based HIV testing venue (RFSL Göteborg, PG Väst)	Electronic	MSM and Trans persons	Aug 13–Nov 30, 2018
Hudkliniken, Centrum för sexuell hälsa	Malmö	Public HIV/STI clinic (Region Skåne)	Paper	All men and women, with integrated MSM-clinic	Aug 20–Oct 31, 2018
Checkpoint Skåne	Malmö	Community-based HIV testing venue (RFSL Rådgivningen Skåne)	Electronic	MSM and Trans persons	Aug 13–Nov 30, 2018

**Fig. 1:**
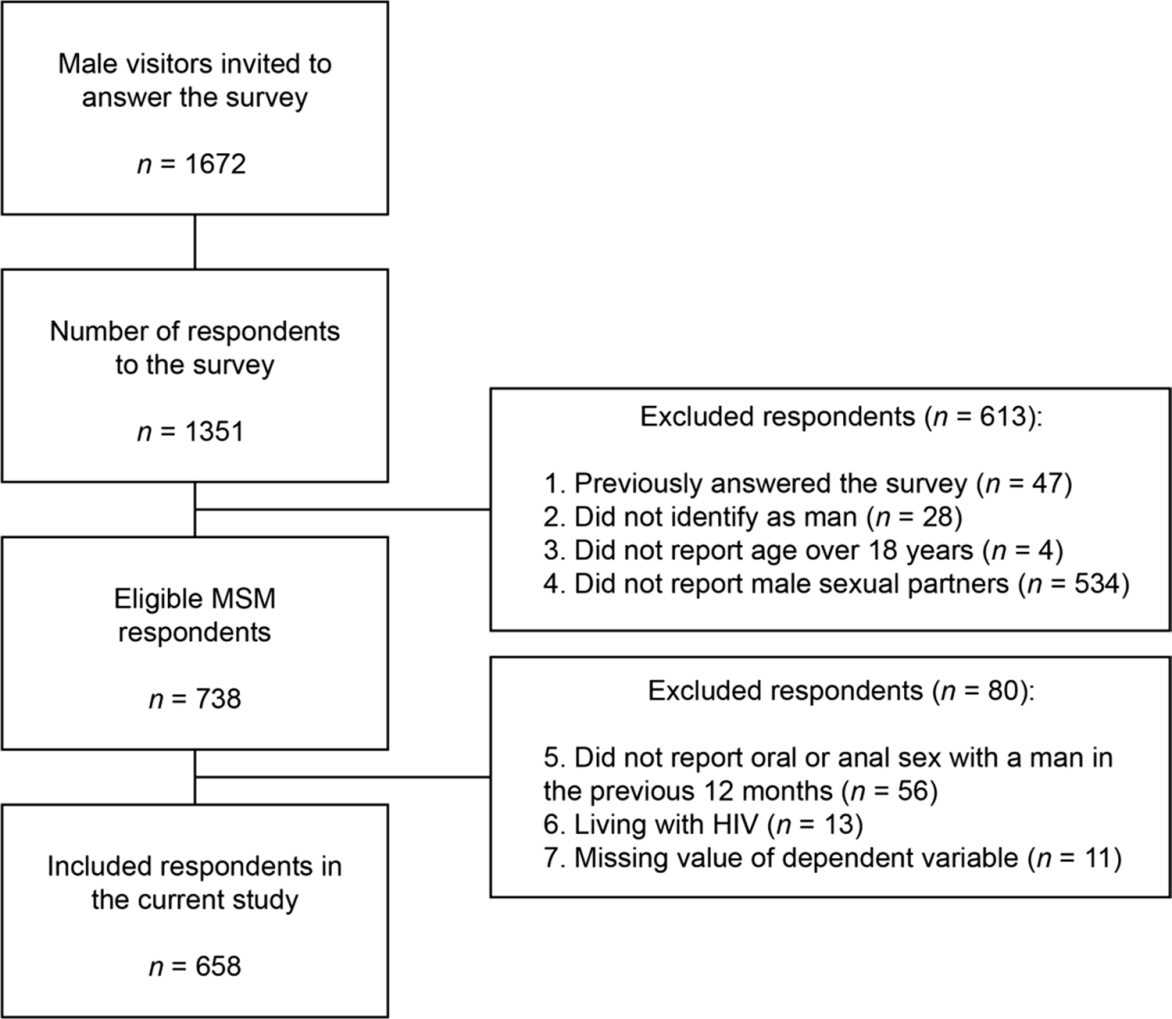
Flow-chart of included respondents

A total of 1672 men were invited to participate in the study. 1351 surveys were submitted, giving a crude response rate of 81%. All men were given the full questionnaire. For the current study, data from 658 participants who met inclusion criteria were analyzed. This selection was based on the following inclusion criteria: identifying as a man, being over the age of 18 years, reported anal or oral sex with another man during the previous 12 months, not having answered the questionnaire previously, not reporting known HIV infection, and no missing value for the main dependent variable. The selection of included participants for this study is illustrated in Fig. 1.

### Measures

The main dependent variable for this study was *Interest in taking PrEP*. Three options were given; “Yes, to take PrEP daily,” “Yes, to take PrEP event-based, around the time of sex,” and “No, I am not interested in taking PrEP/PrEP is not relevant for me.” For the purpose of this study, we chose to dichotomize the variable, merging the two positive replies.

*PrEP knowledge* was assessed by posing four fact statements about PrEP and letting the respondent indicate if they knew this already, by answering either “Yes, I knew this already” or “No, I did not know this already.” The four fact statements were:

1. Pre-exposure prophylaxis (PrEP) involves someone who does not have HIV taking pills before as well as after sex to prevent them getting HIV.
2. PrEP can be taken as a single daily pill if someone does not know in advance when they will have sex.
3. PrEP taken as a daily pill is approved in Sweden and can be prescribed by physicians to people with increased risk of HIV.
4. If someone knows in advance when they will have sex, PrEP can be taken as two pills 2–24 h before sex and then one pill at both 24 and 48 h after the first two pills.

The options were recoded into a new variable that measured the knowledge of the respondents on a five-point scale equivalent to the number of statements the respondent knew, ranging from 0 to 4 statements.

*Self*-*perceived HIV risk* was assessed by posing the question “What do you believe your current risk of getting HIV is?” The options were grouped into three risk levels; “No risk,” “Low risk,” and “Moderate to high risk.”

*Number of male sexual partners* was defined as the number of men the respondents reported having had oral and/or anal sex with during the previous 12 months. The variable was grouped into 1, 2–4, 5–9, and 10 men or more. We also assessed the number of men with whom respondents reported having had receptive condomless anal intercourse (rCLAI), *Number of male rCLAI partners*, during the previous 12 months, was grouped into 0, 1, 2–4, and 5 or more male rCLAI partners.

Three different dichotomized (yes/no) variables for self-reported STIs during the previous year were included: *Any bacterial STI* (Gonorrhea, Chlamydia and/or Syphilis infection, irrespective of anatomical site of infection), *Rectal STI* (rectal Gonorrhea and/or Chlamydia infection), and *Combined high risk STI (*rectal Gonorrhea, rectal Chlamydia and/or Syphilis infection).

*Drug use* during sex was assessed by asking the respondents if they had used any illicit drugs or other means of intoxication in connection with sex during the previous 12 months. Respondents were given the options to reply “No” or, in case of positive replies, to provide specific details on the following intoxicants: alcohol, amphetamine, cannabis, gamma-hydroxybutyrate (GHB) or gamma-butyrolactone (GBL), heroin, ketamine, cocaine, mephedrone, methylenedioxy-methamphetamine (MDMA) or ecstasy, methamphetamine/ice, poppers (alkyl nitrate inhalants), or any other substance, which they were then asked to specify. *Drug use* was defined as use of any of the specified substances excluding alcohol. For the variable *Hard drug use,* we excluded alcohol, poppers, and cannabis.

*Sex abroad* was assessed by asking the respondents if they had sex outside Sweden during the previous 12 months.

### Statistical Analysis

Descriptive statistics presented for all included study participants were used to provide an overview of sociodemographic variables stratified by the dependent variable Interested in PrEP. Differences in proportions between the strata in the descriptive analyses were tested by Pearson’s chi square, and the significance level was set at *p* < .05. The results from this analysis, together with a review of the literature, formed the basis for selection of variables to be include in the logistic regressions. Based on the initial descriptive findings an additional analysis was carried out to assess the accuracy of self-perceived HIV risk by exploring associations between self-assessed HIV risk and risk indicating variables.

As some variables included in the descriptive analysis included partly the same measurements, such as Rectal STI and Combined risk STI, and Number of male sexual partners and Number of rCLAI partners, only one measure was selected for the logistic regressions. Univariable logistic regressions were conducted to obtain crude odds ratios (OR) for the association between the dependent variable and the independent variables regarding risk factors for HIV, self-perceived risk, and knowledge about PrEP, as well as sociodemographic variables. In the full multivariable logistical regression model, only respondents with no missing values for the variables were included. The goodness of fit was tested through a Hosmer–Lemeshow test for the final multivariable model. Data were analyzed using Stata 12.1SE.

## Results

### Participant Characteristics

The included 658 respondents had a median age of 32 years (interquartile range, IQR 27–41). A majority (77.8%) identified themselves as homosexual; 19.0% and 3.2% identified themselves as bisexual and heterosexual, respectively. Seven individuals (1.1%) had been assigned female sex at birth and identified as men at the time of the survey. Overall, respondents had high education levels with 61.6% reporting university education. The majority of the respondents, 63.9%, were born in Sweden, 18.3% were born in another European country, and 17.8% in a country outside Europe. At the time of the survey 60.2% reported being single.

Among the respondents, 68.8% (*n* = 453) were interested in using PrEP. A slightly higher proportion of those interested in PrEP reported being single, i.e., 63.1%, compared to 53.9% among those not interested (*p* = .027). The proportion of men who identified as homosexual was 82.3% among those interested in PrEP and 68.0% among those not interested, while the proportion of respondents who identified as heterosexual or bisexual was higher among those not interested (*p* < .001). The sociodemographic characteristics of the included 658 male respondents in total and stratified for the main dependent variable are presented in Table 2.

**Table 2:** Sociodemographic characteristics of study participants, total and stratified by interest in taking PrEP

Characteristics	Total, no. (%) (*n* = 658)	Interested in PrEP, no. (%) (*n* = 453)	Not interested in PrEP, no. (%) (*n* = 205)	*χ*^2^
Age, years median (IQR)	32 (27–41)	32 (27–40)	33 (27–44)	
Age group				.064
18–25 years	130 (19.8)	90 (19.9)	40 (19.5)	
26–25 years	269 (40.9)	193 (42.6)	76 (37.1)	
36–45 years	137 (20.8)	96 (21.2)	41 (20.0)	
46–55 years	76 (11.6)	51 (11.3)	25 (12.2)	
≥ 56 years	46 (7.0)	23 (5.1)	23 (11.2)	
Sexual orientation				**< .001**
Heterosexual	21 (3.2)	7 (1.6)	14 (6.9)	
Bisexual	124 (19.0)	73 (16.2)	51 (25.1)	
Homosexual	509 (77.8)	371 (82.3)	138 (68.0)	
Sex assigned at birth				.136
Female	7 (1.1)	3 (0.7)	4 (2.0)	
Male	651 (98.9)	450 (99.3)	201 (98.1)	
University education				.198
Yes	402 (61.6)	269 (59.9)	133 (65.2)	
No	251 (38.4)	180 (40.1)	71 (34.8)	
Country of birth				.602
Sweden	412 (63.9)	282 (63.2)	130 (65.3)	
Europe outside Sweden	118 (18.3)	80 (17.9)	38 (19.1)	
Outside Europe	115 (17.8)	84 (18.8)	31 (15.6)	
Relationship status				**.027**
Single	395 (60.2)	285 (63.1)	110 (53.9)	
In a relationship	261 (39.8)	167 (37.0)	94 (46.1)	

Bold font indicate *p* value < .05

As shown in Table 3, the median number of male sexual partners during the last 12 months for the respondents was 7 (IQR: 4–15 partners). The number of male sexual partners was higher among those interested in PrEP as compared to those not interested. The descriptive analysis showed (χ^2^*p* < .05) that among the respondents who were interested in PrEP, there was a higher proportion of respondents with higher knowledge about PrEP, higher self-perceived HIV risk, higher number of male sexual partners and receptive condomless anal intercourse (rCLAI) partners, and more often reported drug use during sex, hard drug use, poppers use, and sex abroad in the last 12 months.

**Table 3:** HIV risk indicators, perceptions and PrEP knowledge among study participants, total and stratified by interest in taking PrEP

Characteristics	Total, no. (%) (*n* = 658)	Interested in PrEP, no. (%) (*n* = 453)	Not interested in PrEP, no. (%) (*n* = 205)	*χ*^2^
PrEP knowledge				**< .001**
0 statements	102 (15.7)	47 (10.5)	55 (27.2)	
1 statement	81 (12.5)	54 (12.1)	27 (13.4)	
2 statements	152 (23.4)	113 (25.3)	39 (19.3)	
3 statements	169 (26.0)	121 (27.1)	48 (23.8)	
4 statements	145 (22.3)	112 (25.1)	33 (16.3)	
Self-perceived HIV risk				**< .001**
No risk	97 (14.9)	42 (9.4)	55 (27.2)	
Low risk	458 (70.5)	325 (72.5)	133 (65.8)	
Moderate to high risk	95 (14.6)	81 (18.1)	14 (6.9)	
Number of male sexual partners, median (IQR)	7 (4–15)	10 (5–20)	4 (2–10)	
Number of male sexual partners, grouped				**< .001**
1 male partner	55 (8.7)	19 (4.4)	36 (18.1)	
2–4 male partners	154 (24.3)	88 (20.2)	66 (33.2)	
5–9 male partners	135 (21.3)	101 (23.2)	34 (17.1)	
≥ 10 male partners	290 (45.7)	227 (52.2)	63 (31.7)	
Number of male rCLAI partners, grouped				**< .001**
0 rCLAI male partner	301 (45.7)	175 (38.6)	126 (61.5)	
1 rCLAI male partner	133 (20.2)	88 (19.4)	45 (22.0)	
2–4 rCLAI male partners	140 (21.3)	118 (26.1)	22 (10.7)	
≥ 5 rCLAI male partners	84 (12.8)	72 (15.9)	12 (5.9)	
Any bacterial STI*				.265
Yes	149 (22.9)	108 (24.2)	41 (20.2)	
No	501 (77.1)	339 (75.8)	162 (79.8)	
Rectal STI^†^				.058
Yes	59 (9.1)	47 (10.5)	12 (5.9)	
No	591 (90.9)	400 (89.5)	191 (94.1)	
Combined high risk STI^‡^				.123
Yes	80 (12.3)	61 (13.7)	19 (9.4)	
No	570 (87.7)	386 (86.4)	184 (90.6)	
Drug use^§^				**< .001**
Yes	271 (41.3)	215 (47.7)	56 (27.3)	
No	385 (58.7)	236 (52.3)	149 (72.7)	
Hard drug use^‖^				**< .001**
Yes	91 (13.8)	77 (17.0)	14 (6.8)	
No	567 (86.2)	376 (83.0)	191 (93.2)	
Poppers use				**< .001**
Yes	229 (34.9)	181 (40.1)	48 (23.4)	
No	427 (65.1)	270 (59.9)	157 (76.6)	
Sex abroad				**.011**
Yes	396 (60.4)	287 (63.6)	109 (53.2)	
No	260 (39.6)	164 (36.4)	96 (46.8)	

Bold font indicate *p* value < .05. *Gonorrhea, chlamydia or syphilis (past year), ^†^rectal chlamydia/rectal gonorrhea (past year), ^‡^rectal chlamydia, rectal gonorrhea or syphilis (past year), ^§^amphetamine, cannabis, GHB/GBL, heroin, ketamine, cocaine, mephedrone, MDMA/ecstasy, methamphetamine/ice, poppers (during sex, past year), ^‖^amphetamine, GHB/GBL, heroin, ketamine, cocaine, mephedrone, MDMA/ecstasy, methamphetamine/ice (during sex, past year)

*PrEP* pre-exposure prophylaxis, *rCLAI* receptive condomless anal intercourse

As self-perceived HIV risk was found to be associated with interest in taking PrEP, further descriptive analyses were carried out to assess the accuracy of this perception among the respondents. The analyses showed that the majority of included risk indicating variables were associated with the self-perceived risk, with the exception of Rectal STI, Hard drug use, and Sex abroad, as illustrated in Table 4.

**Table 4:** HIV risk indicators stratified by self-perceived HIV risk

Characteristics	Total, no. (%) (*n* = 650)	No HIV risk no. (%) (*n* = 97)	Low HIV risk no. (%) (*n* = 458)	Moderate to high HIV risk no. (%) (*n* = 95)	*χ*^2^
Number of male sexual partners, grouped					**.006**
1 male partner	55 (8.8)	14 (14.9)	39 (8.8)	2 (2.3)	
2–4 male partners	152 (24.2)	31 (33.0)	105 (23.7)	16 (18.0)	
5–9 male partners	133 (21.2)	16 (17.0)	96 (21.6)	21 (23.6)	
≥ 10 male partners	287 (45.8)	33 (35.1)	204 (46.0)	50 (56.2)	
Number of male rCLAI partners, grouped					**.014**
0 rCLAI male partner	298 (45.9)	46 (47.4)	221 (48.3)	31 (32.6)	
1 rCLAI male partner	132 (20.3)	26 (26.8)	88 (19.2)	18 (19.0)	
2–4 rCLAI male partners	137 (21.1)	13 (13.4)	96 (21.0)	28 (29.5)	
≥ 5 rCLAI male partners	83 (12.8)	12 (12.4)	53 (11.6)	18 (19.0)	
Any bacterial STI*					**.011**
Yes	144 (22.4)	27 (28.1)	87 (19.3)	30 (31.6)	
No	499 (77.6)	69 (71.9)	365 (80.8)	65 (68.4)	
Rectal STI^†^					.306
Yes	57 (8.9)	11 (11.5)	35 (7.7)	11 (11.6)	
No	586 (91.1)	85 (88.5)	417 (92.3)	84 (88.4)	
Combined high risk STI^‡^					**.034**
Yes	77 (12.0)	14 (14.6)	45 (10.0)	18 (19.0)	
No	566 (88.0)	82 (85.4)	407 (90.0)	77 (81.1)	
Drug use^§^					**.007**
Yes	267 (41.2)	30 (30.9)	187 (40.9)	50 (53.2)	
No	381 (58.8)	67 (69.1)	270 (59.1)	44 (46.8)	
Hard drug use^‖^					.290
Yes	89 (13.7)	10 (10.3)	62 (13.6)	17 (18.1)	
No	559 (86.3)	87 (89.7)	395 (86.4)	77 (81.9)	
Poppers use					**.004**
Yes	227 (35.0)	26 (26.8)	155 (33.9)	46 (48.9)	
No	421 (65.0)	71 (73.2)	302 (66.1)	48 (51.1)	
Sex abroad					.724
Yes	392 (60.4)	55 (57.3)	281 (61.4)	56 (59.0)	
No	257 (39.6)	41 (42.7)	177 (38.7)	39 (41.1)	

Bold font indicate *p* value < .05. *Gonorrhea, chlamydia or syphilis (past year), ^†^rectal chlamydia/rectal gonorrhea (past year), ^‡^rectal chlamydia, rectal gonorrhea or syphilis (past year), ^§^amphetamine, cannabis, GHB/GBL, heroin, ketamine, cocaine, mephedrone, MDMA/ecstasy, methamphetamine/ice, poppers (during sex, past year), ^‖^amphetamine, GHB/GBL, heroin, ketamine, cocaine, mephedrone, MDMA/ecstasy, methamphetamine/ice (during sex, past year)

*rCLAI* receptive condomless anal intercourse

### Variables Associated with Interest in Taking PrEP

In logistic regression analyses (Table 5) we included one measure for STI (Combined high risk STI), one measure for number of partners (Number of male rCLAI partners, grouped), and two different measures of different drug use (Hard drug use and Poppers use). The univariable logistic regression analysis resulted in statistically significant unadjusted associations between each variable explored and interest in taking PrEP, with only one exception, Combined High Risk STI (OR 1.53, 95% CI 0.89–2.16).

**Table 5:** Univariable and multivariable logistic regression for *interest in taking PrEP* in a sample of 658 MSM attending HIV/STI-testing venues in Sweden

Characteristics	Univariable analysis	Multivariable analysis* (*n* = 630)
	Crude OR (CI 95%)	Adjusted OR (CI 95%)
Age group		
18–25 years	**2.25 (1.13–4.47)**	1.60 (0.71–3.59)
26–25 years	**2.54 (1.34–4.80)**	1.76 (0.82–3.75)
36–45 years	**2.34 (1.18–4.64)**	1.86 (0.84–4.17)
46–55 years	2.04 (0.96–4.32)	1.82 (0.74–4.43)
≥ 56 years	1 (ref.)	1 (ref.)
Sexual orientation		
Heterosexual	1 (ref.)	1 (ref.)
Bisexual	**2.86 (1.08–7.59)**	1.63 (0.55–4.80)
Homosexual	**5.38 (2.13–13.60)**	2.29 (0.80–6.60)
Relationship status		
Single	**1.46 (1.04**–**2.04)**	1.26 (0.84–1.88)
In a relationship	1 (ref.)	1 (ref.)
PrEP knowledge		
0 statements	1 (ref.)	1 (ref.)
1 statement	**2.34 (1.28**–**4.28)**	**2.49 (1.22–5.07)**
2 statements	**3.39 (1.99**–**5.78)**	**3.03 (1.61–5.73)**
3 statements	**2.95 (1.77**–**4.93)**	**1.88 (1.01–3.50)**
4 statements	**3.97 (2.29**–**6.88)**	**2.42 (1.24–4.75)**
Self-perceived HIV risk		
No risk	1 (ref.)	1 (ref.)
Low risk	**3.20 (2.04–5.02)**	**3.97 (2.39–6.60)**
Moderate to high risk	**7.58 (3.78–15.18)**	**7.52 (3.52–16.08)**
Number of male rCLAI partners, grouped		
0 rCLAI male partner	1 (ref.)	1 (ref.)
1 rCLAI male partner	1.41 (0.92–2.16)	1.41 (0.86–2.32)
2–4 rCLAI male partners	**3.86 (2.32–6.43)**	**3.26 (1.83–5.81)**
≥ 5 rCLAI male partners	**4.32 (2.25–8.30)**	**4.63 (2.04–10.52)**
Combined high risk STI^†^		
Yes	1.53 (0.89–2.64)	0.76 (0.38–1.50)
No	1 (ref.)	1 (ref.)
Hard drug use^‡^		
Yes	**2.81 (1.55–5.10)**	**2.20 (1.04–4.65)**
No	1 (ref.)	1 (ref.)
Poppers use		
Yes	**2.19 (1.51–3.19)**	1.18 (0.75–1.87)
No	1 (ref.)	1 (ref.)
Sex abroad		
Yes	**1.54 (1.10–2.15)**	1.07 (0.72–1.60)
No	1 (ref.)	1 (ref.)

Bold font indicate *p* value < .05. *All variables included in multivariable model. *p* value for goodness of fit (Hosmer–Lemeshow): .26. ^†^Rectal chlamydia, rectal gonorrhea or syphilis (past year), ^‡^amphetamine, GHB/GBL, heroin, ketamine, cocaine, mephedrone, MDMA/ecstasy, methamphetamine/ice (during sex, past year)

*PrEP* pre-exposure prophylaxis, *rCLAI* receptive condomless anal intercourse

### Variables Independently Associated with Interest in Taking PrEP

In the multivariable logistic regression, all included variables were mutually adjusted for each other. The results of the multivariable logistic regression analysis showed independent associations between interest in taking PrEP and four of the explored independent variables: PrEP knowledge, Self-perceived HIV risk, Number of male rCLAI partners, and Hard drug use. The results revealed that knowing one of the factual statements about PrEP was independently associated with being interested in taking PrEP (AOR 2.49, 95% CI 1.22–5.07), as were knowing two statements (AOR 3.03, 95% CI 1.61–5.73), three statements (AOR 1.88, 95% CI 1.01–3.50), and four statements (AOR 2.42, 95% CI 1.24–4.75). The odds of being interested in PrEP were also higher among those with low (AOR 3.97, 95% CI 2.39–6.60) and moderate to high (AOR 7.52, 95% CI 3.52–16.08) self-perceived HIV risk than among those who reported no perceived HIV risk. With regard to the number of rCLAI partners, the results showed that reporting 2–4 partners (AOR 3.26, 95% CI 1.83–5.81) or 5 or more partners (AOR 4.63, 95% CI 2.04–10.52) was associated with interest in taking PrEP. This association was not found among those reporting only one rCLAI partner (AOR 1.41, 95% CI 0.86–2.32). Reporting hard drug use during sex was also found to be independently associated with interest in taking PrEP (AOR 2.20, 95% CI 1.04–4.65). The Hosmer–Lemeshow test for goodness of fit (10 groups) gave a *p* value of .26, which indicated that the model had a satisfactory goodness of fit.

## Discussion

This study showed that a large proportion of MSM attending HIV testing venues in Sweden expressed interest in taking PrEP for HIV prevention. In line with findings from previous studies conducted in different settings and countries (Aghaizu et al., 2013; Bil et al., 2015; Bull et al., 2018; Frankis, Young, Lorimer, Davis, & Flowers, 2016; Hoagland et al., 2017; Holt et al., 2017; Lee et al., 2017), sampled MSM who reported higher number of partners with whom they had receptive condomless anal intercourse, who reported using drugs in conjunction with sex, and who had a higher self-perceived risk of HIV were more likely to report interest in taking PrEP. We found that the respondents’ self-perceived risk of HIV was a valid risk assessment, as higher self-perceived risk was associated with risk indicators such as number of partners, history of STIs, and drug use. These findings suggest that MSM in Sweden generally have an adequate judgment regarding potential PrEP use in relation to their risk of HIV infection. However, a proportion of the respondents with multiple rCLAI partners still perceived their risk as low or none.

The motivation behind the reported interest in taking PrEP among MSM with low or no self-perceived risk for HIV as found in this study could have several possible explanations, such as high levels of anxiety regarding HIV among some MSM. However, there is a need to examine this further as this might also indicate intention for risk compensation. In a previous study, Golub et al. (2013) found that persons who rated their HIV risk greater also expressed higher willingness to take PrEP, which shows the importance of risk perception for decision-making regarding PrEP use. Golub et al. also raise the issues related to possible risk compensations, i.e., replacing condoms with PrEP. Although results regarding increase of condomless anal intercourse among PrEP users in earlier studies have been inconclusive (Fonner et al., 2016), more recent reviews suggest risk compensation, decreased condom use, and increased incidence of STI among PrEP users (Powell, Gibas, DuBow, & Krakower, 2019; Traeger et al., 2019). PrEP is one strategy to prevent new HIV infections in the subset of MSM who do not use condoms, regardless of their motivations for non-usage. As discussed in an article from 2011, there has been a need to develop prevention programs that include non-condom based prevention strategies toward this group in Sweden (Berg et al., 2011).

A number of recent European studies have raised concerns regarding increased (Glass, Hope, Tanner, & Desai, 2017; Sewell et al., 2018) or prevalent drug use in sexual situations among MSM and its correlation with risks for HIV and STIs (Bourne, Reid, Hickson, Torres-Rueda, & Weatherburn, 2015; Druckler, van Rooijen, & de Vries, 2018; Graf, Dichtl, Deimel, Sander, & Stover, 2018; Hibbert, Brett, Porcellato, & Hope, 2019; Stevens, Moncrieff, & Gafos, 2020; Tomkins et al., 2018). Information on drug use among Swedish MSM is relatively scarce (Folkhälsomyndigheten, 2015; Persson et al., 2016b; Petersson, Tikkanen, & Schmidt, 2016; Rosinska et al., 2018; Strömdahl, Hoijer, & Eriksen, 2019). Data from the MSM2013 survey found that 1% of the respondents had used drugs at the time of their latest sexual encounter (Folkhälsomyndigheten, 2015), and a study analyzing data from the same survey reported higher drug use among men with a broader range of sexual behaviors, including condomless anal sex and group sex (Persson et al., 2016b). Another previous study analyzing data from the European MSM Internet Survey found that “club drug” use during the last 12 months was more common among MSM in Swedish metropolitan areas (8.7%) (Petersson et al., 2016), which might contribute to the comparatively high prevalence of hard drug use in conjunction with sex in our study (13.8%). Although the data are not directly comparable due to differences in sampling and measurements, these findings might be an indication of increasing drug use in conjunction with sex among MSM in Sweden. In line with our findings of higher occurrence of drug use among those interested in PrEP, previous studies have found associations between drug use both with PrEP use and willingness to initiate PrEP (Hibbert et al., 2019; Lee et al., 2017; Sewell et al., 2018).

We also found an independent association between higher knowledge about PrEP and interest in taking PrEP. Although this finding is similar to a number of previous studies from different contexts (Hoagland et al., 2017; Kahle et al., 2018; Lee et al., 2017), an earlier study by Ayala et al.(2013) found that acceptability was associated with less knowledge about PrEP. In our study, the majority of the sampled MSM had some knowledge about PrEP, but the proportion who already knew three or four of the factual statements was limited to 26.0% and 22.3%, respectively. The association between knowledge about and need for PrEP also requires detailed study to ensure that information and knowledge translates into informed decisions about PrEP use among MSM with increased risk of HIV.

An interesting finding was the lack of an independent association between being interested in taking PrEP and self-report of any of the STIs that are currently used as indicators of increased HIV risk among Swedish MSM (Folkhälsomyndigheten, 2017; RAV, 2017). Our finding is in line with a previous study from San Francisco, where patients referred to PrEP use based on STI infection were less likely to be retained in PrEP services, which might be explained by lower motivation for PrEP use and lower HIV risk perception in this group (Hojilla et al., 2018). It is plausible that the time aspect contributed to our finding, but the reasons behind this needs to be further studied. STI history was assessed during the past year, and it is possible that some respondents had altered their behaviors following, for example, a rectal infection and by the time of data collection perceived their HIV risk as low or none based on current sexual behaviors. Also, the parameter “sex abroad” was not significant in the adjusted analysis. However, further exploration is needed in the future, as 74% of new HIV cases among MSM in Sweden in 2018 were reported as having been probably contracted outside Sweden (Folkhälsomyndigheten, 2019).

As there are risks for potential disadvantages of PrEP use in HIV low-incidence settings, e.g., decreased condom use and increased STI incidence (Powell et al., 2019), it is essential that PrEP candidates are also provided with counseling and regular testing for STIs other than HIV. Since this study was conducted, PrEP provision has expanded at several locations in Sweden, and the high interest in taking PrEP found in this study is reflected by the demand at these clinics. This apparent high interest in taking PrEP has had an impact on access, as described in the introduction.

Our study suggests that the real need for PrEP among Swedish MSM could be higher than the estimated figure of 500 (RAV, 2017). However, it is challenging to balance interest in PrEP with actual risk of HIV acquisition in the target population, and there might be a need for some flexibility in relation to clinical indications to meet the self-assessed need for PrEP. Based on this, and the challenge for those prescribing PrEP to identify individuals with actual increased risk of HIV, a likely effect would be some degree of over-prescription, which needs to be taken into account when planning for expansion of PrEP provision. The current situation with support from our findings calls for exploring new strategies in PrEP provision, as access to PrEP has become an issue for MSM with perceived risk of HIV infection in Sweden. Such strategies could include the provision of additional resources that would provide sufficient time for physicians to meet PrEP candidates, which would shorten the waiting period for assessment and prescription. Another strategy that has been suggested is increasing access through task shifting so that assessment and prescription could be provided by trained nurses (O’Byrne, MacPherson, Orser, Jacob, & Holmes, 2019). Whether or not this would be a feasible strategy in a Swedish context needs to be further evaluated, but with the high demand for PrEP there is an evident need for improved access. Yet another strategy that has been implemented by one clinic in Sweden has been to have an online self-assessment tool of HIV risk, similar to that of the HIRI-MSM index (Smith, Pals, Herbst, Shinde, & Carey, 2012). Self-assessment tools might be one strategy used to assist in identify those who are initially interested but with low risk of HIV acquisition. Identifying this group might be of higher importance in a low prevalence setting as Sweden, as the effects of low risk individuals with demand for PrEP might risk overriding the supply and contribute to those at increased risk of HIV not accessing PrEP timely. Such self-assessment tools might also contribute to further improve the accuracy of perceived risk of HIV, which would be of importance for those MSM whose behavior puts them at increased risk of HIV, but who perceive their risk as low and have low interest in PrEP. This group would be less likely to initiate conversations regarding PrEP with their health care provider but is highly relevant for HIV preventive initiatives. Additionally, previous studies showing low self-assessed risk as one of the main barriers to HIV testing among MSM in Sweden (Persson et al., 2016a; Strömdahl et al., 2017), indicate that some of these men are less likely to be assessed through contact with the designated PrEP clinics.

### Limitations

Several limitations need to be considered for this study. The sampled MSM may not be fully representative of Swedish MSM, insofar as they were part of a predominately urban MSM population who seek HIV testing at venues targeting MSM. Furthermore, the study might not have reached at-risk populations with limited access to testing facilities, such as undocumented migrant MSM, men who sell sex, and certain groups of MSM where stigma keeps them from attending MSM-specific venues. Despite a high response rate, we had no information regarding non-respondents, and we can therefore not exclude selection bias in connection to inclusion. Regarding the validity of the data, we need to consider that all information was self-reported, which might have led to some degree of recall bias. Further, as the topics explored in the questionnaire might be regarded as sensitive, there is also a certain risk of social desirability bias, which might have led to under-reporting of behaviors associated with increased risk of HIV and STIs, as well as with self-reported STIs. This might partly explain why we did not find an independent association between self-reported STIs and interest in PrEP. Finally, interest in taking PrEP does not necessarily lead to PrEP use (Rendina, Whitfield, Grov, Starks, & Parsons, 2017; Rolle et al., 2017), but being interested or willing to take PrEP is a pre-requisite for use, adherence, and retention in PrEP care. The extent to which MSM who are interested in PrEP will take PrEP and be retained in PrEP care needs to be further studied in the Swedish MSM population and similar contexts as PrEP provision is expanded over time. Taking the limitations into account, this study nevertheless contributes with new knowledge on PrEP and MSM in Sweden.

### Conclusion

We found that interest in taking PrEP was high among MSM attending HIV testing venues in Sweden. Overall, MSM estimated their risk of HIV acquisition appropriately, and self-perceived risk of HIV was positively associated with interest in PrEP, supporting the use of this measure as an indicator for PrEP prescribers. Men at higher risk of HIV were found to be generally well-informed regarding PrEP, and a majority of MSM with perceived higher risk of HIV also showed interest in PrEP use. These findings, together with experiences from PrEP provision so far in Sweden, support initiatives to strengthen PrEP access for MSM with increased risk of HIV acquisition in Sweden.
